# The Neurotrophins and Their Role in Alzheimer’s Disease

**DOI:** 10.2174/157015911798376190

**Published:** 2011-12

**Authors:** Shelley J Allen, Judy J Watson, David Dawbarn

**Affiliations:** Dorothy Hodgkin Building, School of Clinical Sciences, University of Bristol, Bristol BS1 3NY, UK

**Keywords:** Alzheimer's disease, NGF, BDNF, synaptic plasticity, cholinergic basal forebrain.

## Abstract

Besides being essential for correct development of the vertebrate nervous system the neurotrophins also play a vital role in adult neuron survival, maintenance and regeneration. In addition they are implicated in the pathogenesis of certain neurodegenerative diseases, and may even provide a therapeutic solution for some. In particular there have been a number of studies on the involvement of nerve growth factor (NGF) and brain derived neurotrophic factor (BDNF) in the development of Alzheimer’s disease. This disease is of growing concern as longevity increases worldwide, with little treatment available at the moment to alleviate the condition. Memory loss is one of the earliest symptoms associated with Alzheimer’s disease. The brain regions first affected by pathology include the hippocampus, and also the entorhinal cortex and basal cholinergic nuclei which project to the hippocampus; importantly, all these areas are required for memory formation. Both NGF and BDNF are affected early in the disease and this is thought to initiate a cascade of events which exacerbates pathology and leads to the symptoms of dementia. This review briefly describes the pathology, symptoms and molecular processes associated with Alzheimer’s disease; it discusses the involvement of the neurotrophins, particularly NGF and BDNF, and their receptors, with changes in BDNF considered particularly in the light of its importance in synaptic plasticity. In addition, the possibilities of neurotrophin-based therapeutics are evaluated.

## ALZHEIMER’S DISEASE: AN OVERVIEW

Alzheimer’s disease accounts for about 60% of all dementias, and over 26 million people worldwide are reported to have Alzheimer’s disease [1], and it is likely that nearly double that number will suffer with some form of dementia. Dementia involves a progressive decline in mental function, usually including deficits in memory, language and cognitive processes. It is therefore not just the patients themselves who are affected by the disease but also the millions of carers, often unpaid, who are needed to look after them. Since the greatest risk factor of Alzheimer’s disease is age, the rapidly rising median age of the world’s population may result in a dramatic increase in the prevalence of the disease during the next decade [1]. The impact this will have on global healthcare systems is likely to be devastating. Understanding the underlying processes in the disease and providing new therapies will be paramount. 

**Fig. (1):**
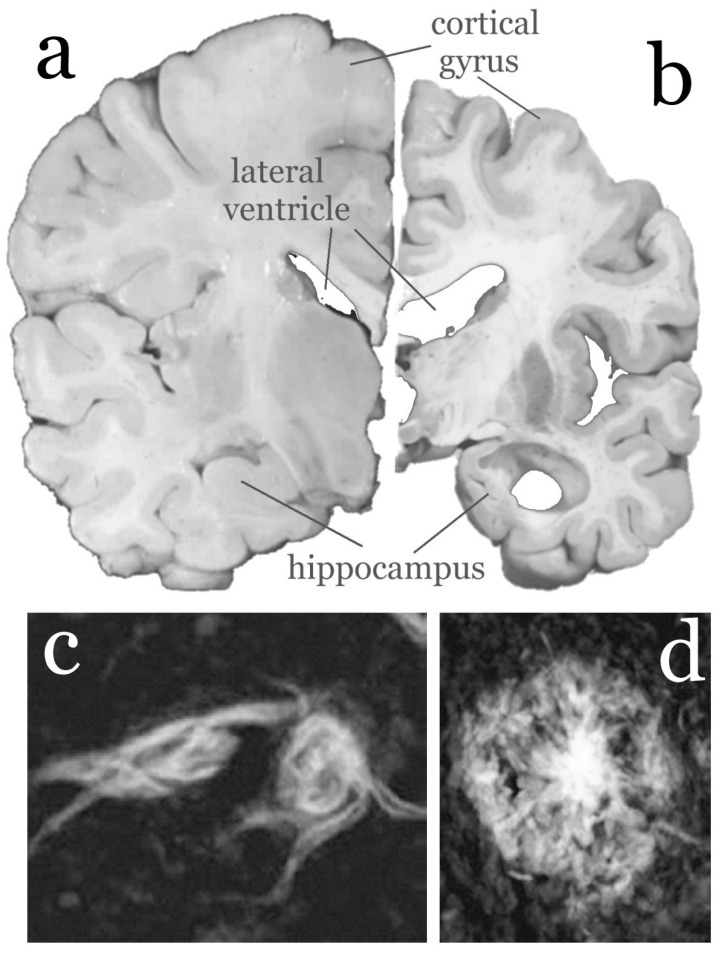
**Gross atrophy of the human brain in Alzheimer’s disease [b] compared with normal [a]**. In moderate to severe Alzheimer’s disease, enlargement of the lateral ventricles is evident, due to cell loss, and thinning of the gyri, producing widened sulci. The hippocampus appears shrunken. Histochemical staining with certain dyes such as Thioflavin S make visible neurons containing neurofibrillary tangles [**c**] and extracellular amyloid plaques [**d**].

In 1907 Alois Alzheimer, a German psychiatrist and neuropathologist, first described the symptoms of short-term memory loss and confusion, and linked it to the neuropathology seen at post-mortem in a 51 year old patient Auguste Deter [2]. The typical pathology of this disease involves gross atrophy of the brain, with thinning of the grey matter in the cerebral cortex, enlarged ventricles indicating neuronal loss, microscopic extracellular amyloid plaques (comprising aggregated amyloid protein), intracellular neurofibrillary tangles (comprised of aggregated tau protein), and cerebrovascular amyloid (amyloid protein surrounding the blood vessels) (see Fig. **1**). 

In Alzheimer’s disease, many areas in the brain have amyloid plaques and neurofibrillary tangles present, especially the frontal, temporal and parietal cortices, the hippocampus, and the cholinergic nuclei of the basal forebrain (ChBF) [3-4]. Amyloid plaques do not seem to have a definite pattern of deposition, perhaps due to the many mechanisms able to clear these extracellular proteins, whereas the deposition of intracellular neurofibrillary tangles seems to follow a well defined pattern [5]. They form first in the transentorhinal cortex, then spread sequentially to the entorhinal cortex, to areas of the hippocampus and then outwards to the cerebral cortex. Numerous studies have indicated that one of the earliest changes in Alzheimer’s disease involves the loss of synapses, which correlates with mental decline [6], eventually leading to marked cell loss throughout a number of brain areas. The symptoms of the disease therefore follow the slow progression of destruction throughout the brain, beginning with the inability to make new memories, a process which is dependent on the hippocampus and its input from the ChBF. 

## THE NEUROTROPHINS: A BRIEF INTRODUCTION TO THEIR IMPORTANCE IN THE HIPPOCAMPUS AND THE CHBF 

The neurotrophins are a family of four similar proteins: NGF, BDNF, NT-3 and NT-4. They all bind with nanomolar affinity to a pan-neurotrophin receptor p75NTR, and each bind separately, with picomolar affinity, to specific tyrosine kinase receptors (Trks): NGF binds TrkA, BDNF and NT-4 bind TrkB, and NT-3 binds TrkC. Further details of their mode of action in development and disease can be found elsewhere in this issue and in reviews [7].

Studies on the importance of the neurotrophins in Alzheimer’s disease pathology focus mainly on NGF and BDNF and their effect on neurons of the hippocampus and the ChBF. The cholinergic basal forebrain neurons express TrkA receptors which respond to NGF by promoting and maintaining synaptic contact with the neurons of the hippocampus and cortex. In the hippocampus BDNF is a vital component of synaptic plasticity and memory formation. LTP (long term potentiation) is a prerequisite for memory formation and is maintained by the action of BDNF at TrkB, which results in structural changes at the synapse. TrkB receptors are also present on some cholinergic neurons and help to support cholinergic function. Thus interruption of expression of either NGF or BDNF, or a change in the levels of TrkA or TrkB receptors, may result in impaired memory formation and neuronal degeneration. Indeed, the expression of both of these trophic factors and their receptors is affected early in the disease process and therefore appropriate neurotrophin-related therapeutic solutions require an understanding of how this happens and how the situation may be remedied.

## THE CHOLINERGIC HYPOTHESIS

In the late 1970s and early 1980s there were reports of considerable deficits in the function of the cholinergic system in post-mortem tissue, including reductions in the activity of choline acetyltransferase, the enzyme responsible for the synthesis of acetylcholine [8-10]. Furthermore, decreased choline uptake and acetylcholine release was reported in biopsy samples from early Alzheimer’s patients [8]. In addition cortical acetylcholinesterase activity has been shown to be reduced [11,12]. The changes in choline acetyltransferase activity and acetylcholinesterase however, are not seen in mild cognitive impairment (MCI) and it may be that they are a result of other changes in cholinergic cells, such as loss of TrkA and reduction of NGF, which have been reported in MCI [13,14]. 

The cholinergic basal nuclei comprising the septal nucleus, diagonal band of Broca and the nucleus basalis of Meynert (nbM), collectively called the cholinergic basal forebrain nuclei (ChBF), provides the major cholinergic innervation to the hippocampus and cerebral cortices [15,16], and thus are important in memory function and attention [17,18]. 

There is a wealth of evidence to show that this cholinergic system is involved in cognitive processes in rats, monkeys, and humans. Lesions of the cholinergic septum and diagonal band of Broca in rats result in a number of memory deficits such as impairment in maze performance [19, 20], and similar lesions affect memory and cognition in primates [21, 22]. Administration of cholinergic antagonists, such as scopolamine, also result in the inability to acquire new information in primates [21] and cause learning impairments in human volunteers [23]. 

The ‘Cholinergic Hypothesis’ of Alzheimer’s disease suggests that this loss of cholinergic function in the ChBF and the cerebral cortex contributes to the deterioration in cognitive function seen in patients with Alzheimer’s disease [24]. Rivastigmine, donepezil and galantamine are routinely administered cholinesterase inhibitors (often abbreviated to CEI) used to counteract this cholinergic deficit. They inhibit the activity of acetylcholinesterase which degrades acetylcholine in the synapse, and are successful at reducing symptoms in approximately half of patients prescribed them. Although improvements in cognition are usually no greater than five points on the ADAS-cog scale, more obvious improvements are noted by carers in behavioural and functional aspects such as attention and social engagement, which can be maintained for four to five years [25]. Unfortunately, their use is ultimately ephemeral and gradually becomes insufficient to maintain the cholinergic signal; presumably as fewer and fewer synapses remain which are able to release acetylcholine. However, recent evidence suggests that memantine, a glutamatergic N-methyl D-aspartate receptor (NMDAR) antagonist may also protect cholinergic function. Memantine, which has been recommended for use in moderate Alzheimer's disease, protects against amyloid-induced loss of basal forebrain cholinergic fibres and the resultant behavioural deficits [26]. This may be *via* a number of neuroprotective mechanisms, and it is possible that a combination of memantine and cholinesterase inhibitors may prove of benefit.

## THE AMYLOID HYPOTHESIS

### APP Processing

**Fig. (2):**
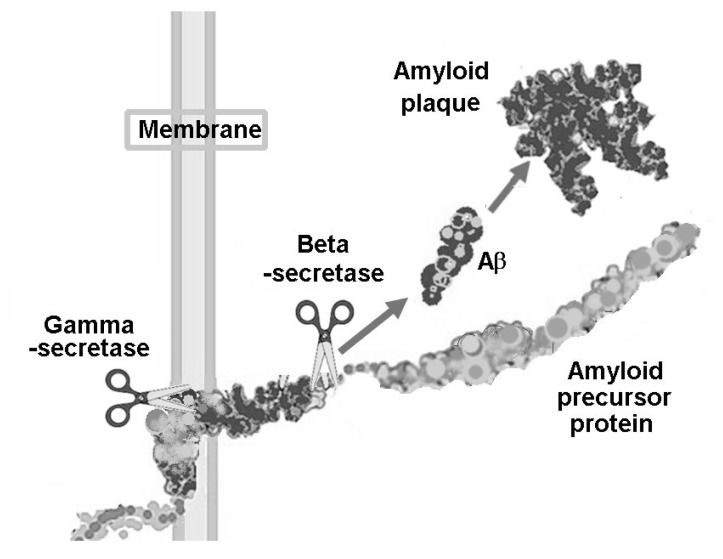
**Production of Aβ by cleavage of APP (amyloid precursor protein)**. Beta-secretase and gamma secretase sequentially cleave APP to form Aβ, which then aggregates to form amyloid plaques.

Despite the fact that the cholinesterase inhibitors are the current mainstay of Alzheimer drug treatment, the majority of new drug targets are not aimed at cholinergic neurons. They are aimed mainly at preventing formation of amyloid or clearing it, or to a lesser extent, preventing or clearing neurofibrillary tangles. Amyloid or Aβ is a 4kDa peptide derived from the larger amyloid precursor protein (APP) by the sequential cutting of two enzymes, beta- and gamma-secretase. Beta-secretase, cloned independently by four separate groups in 1999, is now known as BACE 1 (β-APP site cleaving enzyme 1) [27]. It cleaves the N-terminal portion of APP to leave a 99 amino acid C-terminal portion (C99) which includes the single transmembrane and cytosolic region of APP. Gamma-secretase is now known to be a complex comprised of four proteins including Presenilin 1 (or 2), which has been shown to be the catalytic subunit of the enzyme quartet [28, 29]. It is this enzyme that cleaves APP within the transmembrane region to produce the Aβ peptide (see Fig. **2**). The majority of Aβ produced is 40 amino acids in length (Aβ40), however the site of cleavage may vary and a small proportion of Aβ42 may form, which has a greater propensity to fibrillise. Because of this, the 42 amino acid species usually aggregates into parenchymal plaques, whereas the 40 amino acid form, which is more soluble, is cleared to the blood vessels before it deposits around the vessel cell walls causing cerebrovascular amyloid. 

### Familial Alzheimer’s Disease

The ‘Amyloid Hypothesis’ [30, 31]) proposes that increased Aβ formation, deposition or decreased clearance is sufficient to produce all the neuropathology and associated symptoms leading to dementia in Alzheimer’s disease. This hypothesis followed the discovery that mutations in the APP gene (present on chromosome 21), can lead to an increase in the formation of the Aβ peptide. These mutations are autosomal dominant and therefore families carrying the gene can be traced and studied. Such familial cases usually present symptoms before 65 years and thus are considered to be presenile or early onset. Individuals with Down’s syndrome or Trisomy 21 have a replication of all or part of chromosome 21 (containing the APP gene), and thus an increase in APP gene dosage. It is thought that this is responsible for the finding that Down’s sufferers usually present symptoms of Alzheimer’s disease in their forties. 

Familial Alzheimer’s disease is associated with mutations in one of three possible genes: APP on chromosome 21, PSEN1 on chromosome 14 or PSEN2 on chromosome 1. Mutations in PSEN1, which is the gene coding for the presenilin 1 protein, have been shown to result in an increase in the ratio of Aβ42:Aβ40, by shifting the cleavage site within the transmembrane region of APP. Aβ has been reported as ‘toxic’ to the cell in numerous studies; the reasons given are manifold [32, 33]. Aβ42 is reported to be more noxious than the Aβ40 form, thus an increase in production of the longer peptide is thought likely to accelerate the processes underlying Alzheimer’s disease. This may be exemplified by the finding that some mutations in the PSEN1 gene lead to an onset of symptoms of Alzheimer’s disease patients in their twenties or thirties. 

### Aβ Toxicity and Sporadic Alzheimer’s Disease

The production of Aβ occurs in normal, as well as Alzheimer brain [34]. However, the majority of APP processing in both normal and Alzheimer brain occurs with cleavage by the enzyme alpha-secretase, rather than beta-secretase. Alpha secretase activity is produced by the action of one or more of the ADAMs (A disintegrin and metalloproteinase) family members, most notably ADAM 17 or TACE (TNFalpha converting enzyme) [35]. Thus sequential cleavage of APP by alpha-secretase (instead of beta-secretase) and subsequently gamma-secretase does not result in the formation of Aβ, but a smaller non toxic peptide called p3. 

The symptoms of sporadic (non familial) cases, which are usually late onset and comprise over 95% of cases, may not necessarily result from an increased production of Aβ. These cases may have reduced clearance or some other difference which results in increased sensitivity to the effects of Aβ. Nevertheless, substantial increases in BACE1 activity have been seen in late-onset Alzheimer brain tissue [36], suggesting that there is an increased production of Aβ, at least in some patients. 

Numerous studies have shown that Aβ is toxic to neurons by means of a number of different mechanisms. The brain has a high rate of oxygen consumption and low levels of protective, anti-oxidant enzymes, and thus tends to be vulnerable to free radical damage. Aβ is known to induce production of reactive oxygen species (ROS), which are damaging to cells, and much of the initial damage may occur in the mitochondria. Aβ has been shown to cause an increase in intracellular H_2_O_2_ that disturbs the membrane environment of metabolic pathway enzymes and causes leakage through mitochondrial membranes, and it is the diffusible, oligomeric form of Aβ which is generally thought to be most damaging. In addition, Aβ has been shown to bind to and disrupt a number of receptors [37], including alpha 7 nicotinic acetylcholine receptor, RAGE (receptor for advanced glycosylation end-products), NMDA-R (N-methyl-D-aspartate receptor), low-density lipoprotein receptor-related protein (LRP) and p75NTR [38]. This binding produces a variety of downstream effects, some of which may have deleterious consequences [37].

### Neurofibrillary Tangle Formation

To satisfy the Amyloid Hypothesis, the production of Aβ must also result in an increase in neurofibrillary tangle formation. Neurofibrillary tangles are comprised of an abnormal intracellular aggregation of peptides produced from the cleavage of the protein tau. Tau is a microtubule associated protein which interacts with tubulin and promotes its assembly into microtubules, and also stabilizes them within axons. Phosphorylation of the tau protein may cause it to separate from the microtubules and aggregate into paired helical filaments in the cytoplasm [39]. Analysis of the core protein of these aggregates reveals a 12kDa protein consisting largely of the microtubule binding domains from the middle of the tau protein, cleaved at the N- and C-terminal ends from the full length tau molecule; these eventually form neurofibrillary tangles. The evidence for the ability of Aβ to directly influence the creation of neurofibrillary tangles is sparse; however one of the most convincing studies describes the injection of Aβ42 into the brains of transgenic mice which have a mutation in tau (P301L: which causes neurofibrillary tangle formation in the mice). Less than three weeks after injection there was a fivefold increase in tangle formation, in the area of the amygdala from where neurons project to the injection sites [40]. A proposed mechanism would be an increase in phosphorylation of tau due to the presence of Aβ, resulting in the inability of tau to remain bound to microtubules leading to aggregation within the neuron.

Despite the finding that Aβ production can act as a trigger for neurofibrillary tangle formation and subsequently dementia, there are many researchers who consider the changes in tau as a more important determining factor for dementia. In agreement with this, the extent of neurofibrillary tangle formation in Alzheimer’s disease correlates more strongly with degree of dementia than does amyloid plaque formation [41]. In addition, FTDP-17 (frontotemporal dementia with Parkinsonism linked to chromosome 17; the gene coding for tau is on chromosome 17) does not present with amyloid deposition as a neuropathological feature, yet still produces a profoundly dementing condition with symptoms often appearing before the fifth decade. These include behavioural and personality changes, severe motor difficulties akin to Parkinsonism, and cognitive problems which interestingly, may not affect memory function. 

## NGF AND ITS RECEPTORS IN THE CHBF

### Neurotrophic Factor Hypothesis

In 1981 a unifying hypothesis was proposed for the cause of amyotrophic lateral sclerosis, Parkinsonism, and Alzheimer’s disease [42]. This hypothesized that each of these disorders was due to the lack of a specific neurotrophic hormone. In the case of Alzheimer’s disease a trophic factor required for cholinergic survival would be insufficiently produced by target regions such as the hippocampus, with resulting neuronal loss. In reality, with a disease like Alzheimer’s which progresses over a number of decades, if the loss of such a neurotrophic factor were responsible for neuronal decline then it is likely that this loss would itself be resultant from some other insult or factor which had accumulated gradually. Nonetheless, as with consideration of the relative contributions of amyloid and tau to the disease process, it may be that loss of activity of such a neurotrophic factor may exacerbate effects of Aβ such that the pathology precipitates dementia more readily. Two neurotrophic factor candidates for such a pivotal role in the progression of Alzheimer’s disease are NGF and BDNF. 

### ChBF, NGF and its Receptors 

Subsequent to the hypothesis proposed by Appel, normal brain levels of NGF mRNA were shown to correlate with the degree of ChBF innervation, with the highest NGF and NGF mRNA levels being present in the cerebral cortex and hippocampus [43]. By injection of radiolabelled NGF into the hippocampus or cerebral cortex, NGF was shown to be retrogradely transported from the terminals of ChBF neurons to their cell bodies [44]. NGF interacts with both the pan-neurotrophin receptor p75NTR [45] and the high-affinity NGF-specific receptor TrkA on the surface of the cholinergic neurons [46-47]. p75NGF (later named p75NTR) was the only named receptor for NGF until TrkA was identified in 1991 [48]. p75NTR was shown by immunohistochemistry, in situ hybridisation and radiolabelled binding techniques to be almost exclusively located on neurons of the ChBF in rat, monkey and human brain, with some in the striatum and low levels in the cortex and hippocampus [49-54]. Over 80% of cholinergic neurons were shown to be p75NTR positive. 

Later TrkA mRNA and protein was shown to be present in neurons of the ChBF [55-56]; with over 90% TrkA colocalisation with p75NTR, but with only TrkA not p75NTR expressed in the striatal neurons [56]. In addition ChBF neurons express TrkB or TrkC or a combination of these. It is NGF binding to the TrkA receptor that stimulates signalling pathways, mediating survival and growth effects [46].

### ChBF Neurons in Alzheimer’s Disease

Thus ChBF neurons provide the major source of cholinergic innervation to the hippocampus and cerebral cortex, and these neurons are affected early in Alzheimer’s disease. Cholinergic projections show reduced function and ChBF neuronal cell bodies produce neurofibrillary tangles. In the 1980s a major loss of neurons from the ChBF was reported in Alzheimer brain [57]. Further studies using antibodies to p75NTR, showed relative sparing of the anterior nbM and septal nucleus with most loss being in the posterior part of the ChBF. In older patients (88 years mean age of death) little reduction in NGF receptor positive neurons was noted, although cell shrinkage was evident [58, 59], whereas studies of younger patients reported marked cell loss [60, 61]. This may indicate that patients suffering from a very late onset of the disease may have less cell loss in the ChBF, despite substantial loss of choline acetyltransferase activity in the cortex. 

## NGF AND ITS RECEPTORS IN ALZHEIMER’S DISEASE

### NGF Levels in Alzheimer’s Disease

In the 1980s, in line with the neurotrophic factor hypothesis, the idea emerged that a loss of NGF in Alzheimer’s brain may account for the loss of cholinergic function. However, no change was found in NGF mRNA levels in Alzheimer’s disease cerebral cortex and hippocampus compared with normal brain [62]. Furthermore, measurements of NGF protein in Alzheimer’s disease brain unexpectedly showed either significant or non-significant increases in the cerebral cortex and hippocampus [63-64], although NGF levels in the ChBF were decreased [65]. One explanation for this decrease in ChBF NGF levels might be a failure in retrograde transport NGF from target tissues to the cell bodies. In a mouse model of Down’s syndrome (Trisomy 16), retrograde transport of radiolabelled NGF from the hippocampus to the septum was markedly reduced [66]. As mentioned above, Down’s syndrome sufferers, because of the extra gene copy of APP, invariably present with Alzheimer-type symptoms and pathology. Further studies in this mouse model related this to increased APP protein in the ChBF neurons [67].

### NGF Receptors in Alzheimer’s Disease

The p75NTR receptor has a number of co-receptors, and can act in a ligand dependent or independent manner. In the presence of TrkA it can interact and facilitate an increase in affinity of NGF for TrkA [46, 68]. However, in combination with other receptors, p75NTR is able to induce apoptosis. Thus neuron cultures from p75NTR knockout mice showed reduced sensitivity to Aβ-induced cell death. When crossed with transgenic mice with APP mutations (models of Alzheimer’s disease with amyloid plaque formation and memory deficits), p75NTR knockout mice showed a reduced degeneration in cholinergic neurites despite no reduction in Aβ levels. This suggests that the major pathway by which Aβ-induced cholinergic dysfunction occurs is mediated *via* p75NTR. Furthermore, p75NTR has been shown to bind Aβ, resulting in JNK-mediated apoptosis [38].

An early significant loss has been reported in the number of TrkA-positive neurons in basal forebrain in MCI (46%) and Alzheimer’s disease (56%) compared to normal [69], and this reduction is associated with cognitive decline [70, 71]. In addition, using single cell expression profiling methods individual ChBF neurons showed downregulation of TrkA, TrkB, and TrkC expression during the progression of Alzheimer’s disease from MCI, whereas p75NTR mRNA levels remained stable even in the end stage of the disease. This is in agreement with earlier studies of basal forebrain membranes from Alzheimer’s disease brain in which no difference from normal was seen in p75NTR mRNA levels [72] or in NGF binding at p75NTR receptors [73].

### NGF processing in Alzheimer’s Disease

NGF is produced as a 36kDa precursor protein (proNGF) which is cleaved to the mature form, by furin intracellularly or plasmin extracellularly. As previously mentioned, levels of NGF were unexpectedly found to be increased. However, it had been assumed that the NGF measured in the brain would be in its mature form; unfortunately the antibodies used were able to bind both the mature and the pro-forms, and it was only established much later that in human brain the predominant form is pro-NGF. ProNGF levels have now been shown to progressively increase during the disease process from MCI to end stage, and are associated with poorer performance on cognitive tests [74, 75].

Evidence suggests that cortical cholinergic nerve terminals are dependent on endogenous NGF, and NGF has a role in the continual remodelling of cholinergic associated circuits in adulthood [76]. This leads to the question as to how an increase in proNGF may arise in Alzheimer’s disease, and the consequent effects of this. Investigation of the maturation and degradation of this protein provides some clues. 

Furin and metalloproteases 3 and 7 process proNGF in the transGolgi network (TGN), and it has been generally assumed that proNGF is largely converted to NGF intra-cellularly. However Cuello and colleagues, have provided evidence that the conversion of proNGF to mature NGF is diminished in Alzheimer’s disease, is accompanied by increased degradation of mature NGF, and that this is likely to be an initiating factor in the degeneration of the cholinergic neurons [77, 78]. This theory is supported by the observation of lower levels of plasmin activity in Alzheimer cortex [78]. Following stimulation of cortical tissue, the majority of NGF released would be in the proNGF form, and thus conversion of proNGF to its mature form would occur mainly in the extracellular space. Studies showed that proNGF, plasminogen and tPA are released together following neuronal stimulation, by addition of neurotransmitters or high molarity potassium [77, 78]. Plasminogen is then converted to plasmin by tPA, which in turn converts proNGF to mature NGF. The reduction in plasmin activity results in less cleavage of proNGF to form NGF, and explains the increase in proNGF in Alzheimer’s brain tissue. Furthermore, one recent study finds reduced tPA activity in Alzheimer’s disease cortex but with no change in tPA (tissue plasminogen activator) or plasminogen protein levels. Neuroserpin levels however, were found to be significantly elevated, whilst endogenous plasminogen activator inhibitor-1 and protease nexin-1 levels were unchanged. It seems likely then that neuroserpin inhibition of tPA activity leads to reduced plasmin activity [79].

Any mature NGF which is produced then interacts with its receptors or is degraded by proteases such as matrix metalloproteinase 9 (MMP-9), which under normal conditions is also activated from its pro form by plasmin [78]. Cuello suggests that the apparent disparity can be explained by a corresponding increase in other MMP9 activating factors such as nitric oxide (NO) [78]. NO is produced from inducible NOS (iNOS) which is associated with inflammatory responses, or increased oxidative stress due to ischaemia or hyperglycemia, and is increased in Alzheimer’s disease. An overall increase in MMP9 activity would result in a decrease in endogenous NGF, which would contribute to cholinergic atrophy. A summary of this is in Fig. (**3**). In addition, experiments show that prolonged inhibition of plasmin activity results in reduction in density of cortical cholinergic presynaptic terminals, as measured by vesicular acetyl choline transporter (VAChT) immunoreactivity [78]. 

### ProNGF Signalling 

As mentioned above, signalling through p75NTR is complex due to its many interacting receptors and it has the ability to elicit ligand-dependent and independent, trophic or apoptotic effects [80]. Although p75NTR-mediated apoptosis has been reported following proNGF binding [81], it has recently been shown that proNGF can also have trophic effects by binding TrkA [82-84]. This is with a ten to twentyfold less potency than mature NGF, commensurate with its lower affinity for TrkA [82].

The receptor sortilin has been shown to be a necessary p75NTR co-receptor for proNGF-mediated apoptosis [47]. Sortilin is of the Vps10p protein family, the members of which are important in endocytosis and receptor recycling and trafficking. Sortilin is produced in dendrites, axons and nerve terminals in perinuclear vesicles and the TGN. It is predominantly intracellular, but a small percentage exists at the plasma membrane. The redistribution of sortilin from the lysosome to the cell surface is regulated by the p75NTR co-receptor NRH2, and this has a vital role in making sortilin available for association with p75NTR [85]. Sortilin mediates internalization of proNGF to recycling endosomes, where release of the intracellular domain of p75NTR occurs; this results in nuclear translocation of NRIF (neurotrophin receptor interacting factor), and subsequent cell death [86]. Pro-apoptotic effects through p75NTR can also be *via* JNK activation; for instance through the p75NTR cytosolic region binding NRAGE (neurotrophin receptor-interacting MAGE homolog) [87].

However, no difference in protein levels of sortilin have been reported in hippocampus or basal forebrain from Alzheimer patients compared with normal [82, 88]. Nevertheless sortilin is implicated in age-related neurodegeneration [89] and proNGF has been shown to elicit cell death in basal forebrain slices from aged but not young mice [90, 91]. This has been attributed to increased sortilin levels with age. 

Thus, although there are number of hypotheses surrounding the changes seen in NGF and TrkA, the exact mechanisms, and the order in which they occur, have yet to be determined. 

## BDNF AND SYNAPTIC PLASTICITY 

### BDNF and Long Term Potentiation

BDNF is highly expressed and widely distributed throughout the central nervous system especially in the hippocampus and cerebral cortex [92-93] and is important in the survival and function of hippocampal, cortical, cholinergic and dopaminergic neurons [94-97]. It not surprising therefore that it has been associated with a number of disorders of the brain, including Alzheimer’s disease, Huntington's disease [98], depression [99] schizophrenia [100] and Rett syndrome [101]; with changes in BDNF levels being associated with each of these conditions. 

In addition to maintaining these various neuronal groups, BDNF has another important role: as a key molecule in synaptic plasticity. Long-term potentiation (LTP) is a component of synaptic plasticity, which involves an increase in synaptic strength usually induced by high frequency stimulation [102]. In the hippocampus and limbic structures, LTP provides the cellular mechanism for memory acquisition and consolidation. 

LTP is classically subdivided into early and late forms (E-LTP and L-LTP); BDNF is of profound importance to both forms of LTP [104]. E-LTP requires modification of proteins but not de novo synthesis; BDNF assists induction of E-LTP by acting through TrkB to increase the synaptic response to weak tetanus stimulation [105]. L-LTP, is produced with strong high frequency stimulation, is protein synthesis dependent and is associated with structural changes at synapses [106]. After induction of L-LTP, Ca^2+^ influx occurs through NMDA (N-methyl-d-aspartate) receptors or voltage-gated Ca^2+^ channels leading to secretion of BDNF [107]. BDNF then binds to TrkB localized at pre- or postsynaptic glutamatergic synapses [108]; at postsynaptic sites, TrkB associates with NMDA receptors and PSD95 (post synaptic density 95) [109]. 

Removal or reduction of BDNF, using BDNF antisense [110, 111], anti-BDNF antibodies [112] or in BDNF heterozygous knockout mice [113-115] produces a marked deficit in L-LTP. Furthermore, BDNF heterozygous knockouts have a reduction in hippocampal axon collaterals and varicosities [116] and profound spatial memory and learning deficits. L-LTP can be rescued in these mice by viral transfer of BDNF [117], even when all new protein synthesis is blocked [114], suggesting that it may be the only protein required to maintain L-LTP. Likewise TrkB is required for L-LTP; anti-TrkB treatment in hippocampal slices inhibits L-LTP [118], and loss of the full length catalytic form of TrkB, as in the conditional TrkB knockout mice, results in severe impairment of learning [119, 120].

### ProBDNF and Long Term Depression

Induction of LTP results in the increased formation of synapses at dendritic spines or axon collaterals [121]. Conversely, long term depression (LTD) entails a reduction in synaptic strength, and is usually induced experimentally by low-frequency stimulation [122]. Whereas BDNF and TrkB, but not p75NTR, are important in LTP [114], proBDNF, *via* binding at p75NTR, is important in LTD [114, 123, 124]. There are contradictory studies as to the extent of processing of proBDNF to BDNF, whether this occurs intracellularly or extracellularly, by which protease, and whether proBDNF can remain unprocessed long enough to have a role in LTD [125-127]. This may partly depend upon where BDNF mRNA is situated within the neuron. For instance, transcripts of BDNF are polyadenylated at two alternative sites producing a short and long version of 3′ UTR, which are involved in different cellular functions. In hippocampal neurons, the short 3′ UTR mRNAs are restricted to soma, whereas the long 3′ UTR mRNAs are also localized in dendrites, where they are important for dendritic pruning. 

### BDNF Processing and Regulated Secretion 

**Fig. (4):**
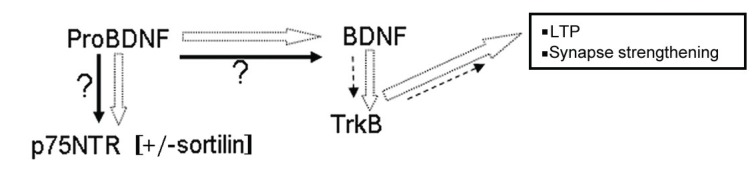
**ProBDNF and BDNF interactions with receptors**. In normal brain (white arrows), proBDNF is processed to BDNF extracellularly by plasmin, or intracellularly by furins or proconvertases. BDNF acts at TrkB facilitating LTP. This leads to synapse strengthening. In Alzheimer brain (arrows in black) proBDNF is downregulated (perhaps directly due to Aβ), thus BDNF levels are reduced. This will lead to a reduction in LTP and synapse formation. Length and thickness of arrows denotes strength of activity. Question marks denote unknowns.

The processing pathway of BDNF is therefore extremely important in determining the outcome of synaptic remodelling. ProBDNF is processed to BDNF by furin within the endoplasmic reticulum or proconvertases in secretory vesicles, or extracellularly by plasmin; thus mature BDNF is then able to bind TrkB facilitating LTP (127; Fig. **4**). Whereas proNGF, proNT-3 and proNT-4 are all largely processed *via* a nonregulated secretory pathway, proBDNF is able to be sorted to a regulated secretory pathway [128-129], through interacting with the sorting receptor carboxypeptidase E [130]. BDNF has a motif (Ile16, Glu18, Ile105, Asp106) within the mature protein [130], mutation of which resulted in mis-sorting of pro-BDNF to the constitutive pathway. Thus mutated BDNF was located mostly in the perinuclear region, whereas wild-type BDNF occurred in a punctuate manner along cell processes. Conversely, when NGF was mutated to contain this sorting motif, it became partially redirected to the regulated pathway [130]. Furthermore, carboxypeptidase E knockout mice showed no activity-dependent secretion of BDNF in cortical neurons [130], although mature BDNF was released *via* the constitutive pathway.

In addition to the motif within the mature region which interacts with carboxypeptidase E, BDNF also has a motif within the prodomain which affects secretion [131]. Sortilin was shown to interact with proBDNF *via* this motif and to colocalize with proBDNF in secretory granules in neurons. Down regulation of sortilin by small interfering RNA (siRNA) in primary neurons led to proBDNF missorting to the constitutive secretory pathway. Thus the presence of a common BDNF polymorphism in the pro-region of proBDNF (Val66Met; 19-45% in varying populations [132]), in the region of the sortilin binding site, resulted in impaired regulated secretion of BDNF after potassium depolarization [133]. Cultured hippocampal neurons transfected with the Met66 proBDNF did not show the Val66-associated proBDNF colocalization with synaptic markers in dendritic processes, but showed perinuclear clustering, indicating that proBDNF sorting from the Golgi complex was affected [133]. Subjects carrying this polymorphism have been shown to be less successful in tests of episodic short-term memory, and functional MRI (magnetic resonance imaging) shows differences in glucose utilization [133, 134]. They also have a smaller hippocampus and prefrontal cortex [135]. 

The sorting of proBDNF for regulated secretion therefore probably involves primary interaction between the pro-domain and sortilin, which promotes an appropriate protein conformation of proBDNF. The sorting motif in the mature domain of BDNF subsequently interacts with carboxypeptidase E, and sorts BDNF into the regulated secretory pathway. The prodomain may therefore be necessary, but not sufficient to sort BDNF into the regulated pathway. ProNGF, despite having no sorting motif in the mature domain, which prevents it from entering the regulated secretory pathway, has the required binding motif to enable binding to sortilin. This promotes correct folding during maturation, but also facilitates its internalisation *via* sortilin and p75NTR to elicit intracellular signalling. 

## BDNF, ALZHEIMER’S DISEASE AND DEPRESSION

### BDNF and Depression

It has been noted that a large percentage of Alzheimer patients suffer from depression, and moreover that severe depression can sometimes produce symptoms similar to early Alzheimer’s disease. Serotonergic pathways are markedly affected in Alzheimer’s disease and this may account to some extent for the associated depression. Reductions in serum BDNF levels have been reported in patients with depressive disorders [136], whereas increased BDNF levels are seen following antidepressant treatment [137]. It is known that the expression of BDNF is trophic for serotonergic neurons [138], and that serotonin stimulates BDNF production. This is in accordance with the finding that SSRIs (selective serotonin uptake inhibitors), commonly used as antidepressants, increase brain BDNF levels. BDNF mRNA levels in the hippocampus have also been shown to be significantly increased with physical exercise [139] and electroconvulsive therapy [140], which is sometimes used as a treatment for depression. The influence of the Val66Met polymorphism on depression is difficult to assess in the light of the disparity of results from a number of studies. However, in general, there appears to be some relationship between the presence of this polymorphism and likelihood of depression or recovery with antidepressants [141].

### BDNF and Alzheimer’s Disease

It has been suggested that the early memory dysfunction seen in Alzheimer’s disease may be related to the levels of BDNF in the hippocampus. A summary of this is in Fig. (**4**). Evidence to support this includes substantially reduced BDNF mRNA levels in Alzheimer’s disease hippocampus [142] and parietal cortex [143] and decreased protein levels of BDNF in entorhinal cortex, hippocampus, temporal, frontal and parietal cortex [144-147]. Unlike mature NGF, mature BDNF protein can be visualised by western blotting, together with its pro-form. Both forms have now been shown to be reduced in Alzheimer’s disease, with a reduction in mature BDNF of 23% reported in frontal cortex [146]. Furthermore a progressive decrease from normal was seen in proBDNF in MCI (21%), compared with Alzheimer (30%) parietal cortex [148-150]. 

The changes in BDNF levels however, seem to be due to specific downregulation of certain BDNF transcripts. There are at least seven BDNF transcripts expressed in human brain, and a number of splice variants of these exist [150]. Only three of these transcripts have been shown to be down-regulated in Alzheimer’s brain (I, II, and IV (previously named III)). In a study using the human neuroblastoma cell line (SH-SY5Y) addition of Aβ42 resulted in down-regulation of BDNF, specifically through BDNF transcript IV, the most abundantly expressed BDNF transcript in human cortex [151]. This downregulation may be due to the effect of Aβ on CREB phosphorylation. Phosphorylation of CREB (transcription factor: cAMP response element-binding) enables binding to CREB-binding protein (CBP) resulting in stimulation of CRE-dependent gene expression. Increased Aβ levels cause downregulation of CREB phosphorylation with a resultant reduction in CRE-dependent gene expression, including BDNF transcription [152]. 

The consensus of a number of studies on the possible effect of the Val66Met polymorphism in Alzheimer’s disease is that the presence of the Met66 allele is a risk factor, and is associated with increased progression to Alzheimer’s disease [153, 154]. In addition, other BDNF polymorphisms may also be implicated [155]. 

Changes in neurons expressing the BDNF receptor TrkB, have also been found in Alzheimer’s disease. Thus, in post-mortem tissue a 47% reduction in TrkB positive neurons has been reported [156], which may be due to a loss of neurons or a downregulation of TrkB expression. In addition a selective reduction in the TrkB protein catalytic form (145 kDa), but not the non catalytic form (95kDa), was found in both frontal and temporal cortex in Alzheimer’s disease [157]. The non-catalytic form is present on a large number of different cell types in the brain, many of which may not be affected in Alzheimer’s disease.

In summary, there is a marked reduction in BDNF levels in both the cortex and ChBF in Alzheimer’s disease, and this may have a bearing on support for cholinergic neurons as well as a deficit in LTP with wide significance for synaptic plasticity in a number of different neurons. With the likelihood that the Val66Met polymorphism is a risk factor for progression of the disease, we are perhaps moving one step closer to personalised therapeutics and to pre-selection for drug trials.

## THERAPEUTIC SOLUTIONS ASSOCIATED WITH NGF AND BDNF

### *In Vitro* and *In Vivo* Animal Studies Associated with NGF

NGF has been shown in a large number of studies to increase survival and function (often measured by choline acetyltransferase activity) of ChBF neurons both *in vitro* [158-161] and *in vivo* subsequent to cholinergic lesion. Lesioning of the fimbria-fornix in rats is a method routinely used to produce deficits in the ChBF. This procedure prevents cholinergic terminals in the hippocampus from being able to retrogradely transport NGF back to the cell bodies in the septal nucleus. This results in atrophy and some loss of cholinergic neurons. Infusing NGF into the cerebral ventricles, adjacent to the ChBF nuclei, prevents this degeneration [162-164] and reverses behavioural deficits such as impairment in water maze performance [163-165]. Interestingly, some aged animals have also been shown to have reduced cholinergic function, and cerebroventricular infusion of NGF in this subpopulation of aged rats normalises their behaviour [163, 166-168].

Heterozygous NGF knockout mice have reduced levels of NGF in the hippocampus, display memory impairment, show shrinkage and loss of cholinergic septal cells and have decreased cholinergic innervation of the hippocampus. Long term infusions of NGF (for five weeks) into the lateral ventricles was shown to abolish the memory deficits and correct the size (although not number) of cholinergic neurons, and also to increase the density of cholinergic innervation of the hippocampus [169].

### An Anti-NGF Model of Alzheimer’s Disease

One of the problems with the classic animal models of Alzheimer’s disease (e.g. APP mutations) is that they do not reflect all of the changes see in Alzheimer’s patients, such as loss of cholinergic function or the production of tau pathology. In 2001 a transgenic mouse was produced as a model of cholinergic deficit. Since NGF knockout mice have deficits related to development, a mouse was engineered which produced antibodies to NGF which accumulated with increasing age. These mice (named AD11) showed a reduction in cholinergic cells staining for choline acetyltransferase in the basal forebrain and exhibited difficulty in executing memory tasks. However, unexpectedly they also showed cortical cell loss, and the presence of amyloid plaques and hyperphosphorylated tau in the cortex and hippocampus [170], suggesting that loss of NGF in adulthood may be sufficient to produce Alzheimer-like neuropathology.

Intranasal administration of NGF restored cholinergic function and reduced the number of amyloid plaques and hyperphosphorylated tau deposits [171]. Intraperitoneal injection of galantamine also restored the number of choline acetyltransferase-immunopositive neurons to normal and reduced numbers of amyloid plaques, although this drug was unable to reduce deposition of hyperphosphorylated tau [171].

### Phase I Trial: Infusion of Mouse NGF

In the 1990s a small Phase I trial was carried out in Sweden in which purified mouse NGF was infused intra-cerebroventricularly [172, 173]. In two patients 6.6 mg NGF was infused over three months. The third patient was infused with three doses of NGF (<0.25 mg) given separately for a total of 22 weeks, for a period of more than a year. The patients were tested cognitively, with magnetic resonance imaging (MRI), positron emission tomography (PET) and electroencephalography (EEG). (^11^C)-Nicotine binding was used as a measure of cholinergic synapses. The first two patients showed slight improvements in cognitive testing; one of these showed a marked increase in frontal and temporal cortical blood flow that continued for several months after the NGF treatment had ceased; they also had an increase in (^11^C)-nicotine binding in the same regions and the hippocampus. One of these also showed improvement in glucose metabolism. The third patient showed less improvement although increased (^11^C)-nicotine binding was observed in the hippocampus seven months after the end of the second period of NGF treatment. 

However, after 11 days of treatment, the patients complained of lumbar pain, which ceased within two days of stopping the treatment. Subsequent studies in rats and primates showed induction of Schwann cell hyperplasia [174]; recombinant human NGF was administered intra-cerebroventricularly to rats for 12 weeks, and tissue sections were then taken from the region around the surface of the medulla and the spinal cord. These showed the presence of adherent non-malignant hyperplastic tissue formed from proliferating Schwann cells, which stained for p75NTR but not TrkA [174]. After stopping the NGF infusion, the hyperplasia gradually reduced in size and disappeared after a number of weeks. A second study in which recombinant human NGF was infused into both rat and primate cerebral ventricles showed that hyperplasia was produced in a dose-dependent fashion [174]. 

Clearly, this hyperplasia had arisen because of the high levels of NGF infused into the ventricles. Other means of providing NGF to the brain were then developed.

### Phase I Trial: Implantation of NGF-Producing Fibroblasts

In 2001 a Phase I trial of eight early stage Alzheimer’s patients assessed the effect of implantation of fibroblasts, modified to produce NGF, into the basal forebrain [175]. Mark Tuszynski and colleagues at the University of California took fibroblasts from each patient, cultured and transfected them with a retroviral vector directing the cells to secrete human NGF before implantation. Unfortunately, two of the patients were severely affected by the intrusive surgical procedure, one of whom died; in the six remaining patients no undesirable effects were seen for up to 22 months post-treatment [175]. MMSE scores, which were on average declining at a rate of six points per year, slowed to three points per year, equivalent to a slowing of the disease by 36–51%. PET analysis of four subjects (those given a higher cell count at implantation) when tested between six and eight months after implantation, showed a striking increase of fluorodeoxyglucose uptake, to areas receiving cholinergic input from the ChBF. In 2008 following examination by autopsy of four people from the trial, Tuszynski noted that each subject exhibited a clear increase in cholinergic cell growth in response to NGF, and concluded that “additional clinical trials of NGF for Alzheimer’s disease are warranted” [176]. 

### Further Trials of NGF

Whilst problems had been encountered with administration of NGF, other methods of delivery have been devised. Ceregene Inc. (San Diego) successfully carried out a Phase I study to assess adeno-associated virus (AAV) mediated delivery of NGF (CERE-110; AAV2-NGF). CERE-110 is an AAV serotype 2-based vector producing human NGF administered by stereotactic injection to the nbM [177]. A USA based multicentre Phase II clinical trial has now begun recruiting patients for a randomized, controlled study in up to fifty subjects with mild to moderate Alzheimer's disease, with half of the patients due to undergo a placebo surgery with no medication injected. 

### *In Vitro* and *In Vivo* Animal Studies Associated with BDNF

BDNF administration into the entorhinal cortex in animal models of Alzheimer’s disease recently produced promising therapeutic results [178]. The entorhinal cortex is one of the first areas affected in Alzheimer’s disease; TrkB receptors are expressed there and it is a major provider of BDNF through its input to the hippocampus. In transgenic mice with APP mutations, lentiviral vectors expressing BDNF were injected into the entorhinal cortex, an area with amyloid plaques and cell loss. This did not affect either the neuronal number or amyloid plaque density; yet recovery of synapses and improved cell signaling was seen, together with amelioration of spatial memory deficits. Similarly in aged rats (24 months old), bilateral, 28 day infusions of BDNF improved spatial learning and memory in water maze performance, and restored cell signaling. Furthermore, improvements were also seen in aged primates, where BDNF gene delivery to the entorhinal cortex reversed neuronal atrophy and ameliorated age-related cognitive impairment. The authors point out that this approach could be scaled up to deliver BDNF to the human entorhinal cortex, and that the “potency of BDNF seen in the preclinical models provides a rationale for exploring clinical translation” [178].

In addition, since SSRI antidepressants are known to elevate BDNF levels, research and clinical trials are being carried out to investigate the effect of these in Alzheimer’s disease; one such antidepressant, Citalopram, is currently undergoing Phase II clinical trials. 

### Small Molecule Therapeutics

Future directions in NGF and BDNF-based therapies may target the neurotrophin receptors. Modulation of p75NTR by a small molecule ligand has already been suggested as a way of preventing p75NTR-mediated cell death. LM11A-24 and -31 are small molecules which block the effect of this receptor with low nanomolar potency, and are reported to prevent Aβ induced cell death and inhibit tau phosphorylation [179]. Derivatives of these compounds have been described as candidates for clinical development. In addition small molecule mimetics of NGF or BDNF have also been suggested as Alzheimer therapeutics [180, 181]. Using *in* *silico* screening with a BDNF loop–domain pharmacophore, Longo and colleagues have identified small molecules with nanomolar neurotrophic activity at TrkB, which prevented neuronal degeneration and improved motor learning after traumatic brain injury in rats. These are being taken forward as lead compounds for future treatments [181].

## SUMMARY

The neurotrophins NGF and BDNF are associated with the early changes seen in MCI leading on to Alzheimer’s disease. Notably an increase in proNGF is seen in the cerebral cortex, together with a loss of Trk receptors in the ChBF neurons, with no change in p75NTR or sortilin receptors. In conjunction with this are reductions in both BDNF and proBDNF in the cortex and hippocampus. There are various theories as to how this occurs and how it affects the progression of the disease. Firstly, it is likely that Aβ is the initiating factor; it is able to bind to a number of receptors, has significant deleterious effects in the mitochondria, and is able to modify gene expression. However, the specific effects on the neurotrophins and their receptors are important at the site most vulnerable early in the disease, the hippocampus, and may be crucial in the development of the disease. 

A model of Down’s syndrome, itself a “model” of Alzheimer’s disease, has virtually no retrograde transport of NGF to the ChBF cell bodies, and it is possible that likewise there is a defect in retrograde transport of NGF in Alzheimer’s disease. This would account for the low level of mature NGF in the cholinergic cells. Another hypothesis gaining credence is that the processing of proNGF to NGF is impaired, due to a decrease in the activity of plasmin, a protease which cleaves proNGF. It is interesting also that plasmin probably helps to regulate Aβ levels in the brain, along with other enzymes such as neprilysin and insulin-degrading enzyme. An increase in MMP9, perhaps due to the increased NO levels seen in Alzheimer’s disease, may then give rise to an increase in the degradation of NGF. 

In parallel with this, or perhaps because of this, there is a loss in Trk receptors on the ChBF neurons, even in MCI. Currently one favoured theory is that p75NTR signalling in response to proNGF, whether pro-survival or pro-apoptotic may depend upon the ratio of TrkA /p75NTR in the ChBF and NGF/proNGF levels produced in the target tissues. Thus the decrease in TrkA, with no change in p75NTR or sortilin levels, will alter the outcome of proNGF effects on the neuron, promoting pro-apoptotic rather than trophic events.

Current NGF-related therapeutic intervention continues with clinical trials using viral transfer of NGF-producing capability into the brains of Alzheimer’s patients, and alongside is the possibility of small molecule intervention at TrkA or p75NTR.

The change seen in BDNF levels does not seem to reflect the same pattern as that of NGF. There is a reduction in proBDNF as well as BDNF and this may be due to the difference in processing of proBDNF compared with proNGF. However the downregulation of BDNF may be at a gene level and may be connected with interference by Aβ with CREB. The loss of BDNF, considering its importance in synaptic plasticity, may explain the reduction in synapses seen in early stage Alzheimer’s disease. 

As with NGF, BDNF therapy in animals by infusion or viral transfer seems to successfully ameliorate cognitive impairment. It is expected that this will eventually be translated into a clinical setting. The possibility of using small molecule TrkB agonists is also being investigated.

It may be that NGF or BDNF-associated treatment will be important in the future as early stage therapeutics for Alzheimer’s, whether or not the initial cause is neurotrophin related. However, in order to provide the most effective and sustainable treatment, there must also be an understanding of what is happening at a molecular level before even the first symptoms appear, and the role that neurotrophins and their receptors play in this.

## Figures and Tables

**Fig. (3):**
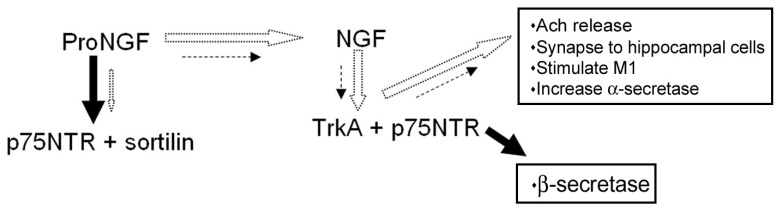
**ProNGF and NGF interactions with receptors**. In normal brain (white arrows), proNGF is processed to NGF extracellularly by plasmin, and intracellularly by furins. NGF acts at TrkA, facilitated by p75NTR. This leads to acetylcholine release and activation of M1 muscarinic receptors which leads to increased alpha-secretase activity, which is counter to Aβ production. In Alzheimer brain (arrows in black) proNGF is not processed properly to NGF. The increased level of proNGF will lead to increased binding at p75NTR and probably sortilin, leading to a greater likelihood of cell death. Due to an increase in MMP9 activity NGF is degraded more quickly. Thus less acetylcholine will be released, less communication with other neurons, less activation of M1 receptors and an increase in beta-secretase activity. The latter will lead to an increase in Aβ formation. Length and thickness of arrows denotes strength of activity.
